# Progress in Surgery

**Published:** 1899-01-07

**Authors:** 


### PROGRESS IN SURGERY.

### RHINOLOGY.

(*Continued from page* 233.)

**Nasal Hydrorrhœa.**—The clinical picture of nasal hydrorrhœa shades off in one direction into cases of what are generally called hay fever, with symptoms of intense local irritation; while in the other direction they may consist of a passive and almost painless watery discharge from the nose. It appears to be an affection of adult life affecting males and females indifferently. Although it may be more marked on one side than on the other, the flow usually takes place from both nostrils. When handkerchiefs are soaked with it they generally dry stiff. In cerebro-spinal rhinorrhœa, on the other hand, the discharge is so watery that handkerchiefs dry quite soft, and can be used again without washing; and in this affection the discharge is limited entirely to one nostril, unless there happens to be some obstruction on the affected side, when it may make its way round to the opposite nasal fossa. When the fluid is of arachnoid origin there is frequently headache or other mental symptoms, which are relieved by the discharge. It is not accompanied with lachrymation or suffusion of the conjunctiva, and photophobia; and although it may occasionally give rise to a little sneezing—especially in the morning, or on rising—this is a rare and infrequent accompaniment.

In nasal hydrorrhœa, feelings of *malaise* set in with the discharge and only disappear with its cessation. It is frequently ushered in with sneezing, photophobia, lachrymation. It rarely continues in sleep, while cerebro-spinal rhinorrhœa continues day and night. It is very erratic in its onset and in its intermission, and it is very dependent on external influences and on conditions of health. Moritz Schmidt states that some cases have been observed which were dependent on ulcer of the stomach or biliary lithiasis; and while using the term which forms the title to this paper, he defines the disease as a vasomotor rhinitis. McBride recognises the diversity of the conditions of which nasal hydrorrhœa may be but a symptom.

On the question of treatment Thomson utters a warning against interference from the risk of septic infection whenever there is any reason to suspect that the fluid is of cerebral origin. The only treatment is empiric, and on the lines adopted for hay fever, but he enters a plea for moderation in the use of the galvano-cautery. Careful general treatment, hygienic, dietetic, and climatic, with possibly a visit to a suitable spa, will frequently secure very satisfactory results. Mr. Cresswell Baker, in the course of a discussion, instanced a case in a lady. The discharge was clear and watery from one side of the nose, persisting day and night. He collected 70 minims in five minutes. The interesting point was that, after trying various kinds of treatment, he applied the continuous current externally to the two sides of the nose; the first application arrested the discharge in a quarter of an hour, then the patient applied it herself, and the discharge ceased gradually. Three months afterwards there was no return. Dr. Middlemass Hunt cited another case in his practice where a girl lost her rhinorrhœa on returning home and discontinuing work. He thought it had arisen from a general lowering of health.

**Blue Nasal Secretion.**[14]—At the annual Congress of the French Society of Otology and Laryngology, M. Molinié, of Marseilles, related the case of a young woman, 25 years of age, in whom, after a severe attack of grippe, accompanied by intestinal, gastric, and nervous symptoms, there occurred a discharge of blue secretion from the nose. In the beginning the discharge was generally viscous and colourless. Several times during the day, however, the mucus was streaked by lines of blue as deep as methylene blue. Examination of the nasal fosse demonstrated that the source of the secretion was the middle meatus of the right side. Bacteriological investigation revealed the presence of a short, squat bacillus with rounded extremities, coloured by methylene violet and gentian violet, and retaining its colour under the Gram reagent. Although cultures did not yield the characteristic blue colour, it is very probable that this case of blue chromorhinorrhœa was due to the development of a pyocyanic colony in the frontal sinus of the right side.

**Nasal Fibroma.**—Casselberry reports again a case of nasal fibroma which he had recorded ten years previously, adding a subsequent clinical report and a full pathological report. The growth, which was of considerable size, producing external deformity, was attached to the horizontal plate of the ethmoid bone corresponding to the middle turbinated bone, which had been absorbed. Eleven years later it had not recurred. Dr. J. Wright, in reporting on the growth, admitted that it might be termed a fibroma, although there were a number of œdematous areas in it as well as evidences more commonly regarded as those of chronic inflammation. He said the longer he studied nasal pathology the harder he found it to draw the line between inflammatory and true benign tumours, and the more inclined he is to believe that that line is a very arbitrary one and really should have no existence, as benign neoplastic growths are, he believes, all the result of chronic inflammation or analogous metabolic processes.

**Suprarenal Gland Extract.**—W. H. Bates[15] has been using the aqueous extract of the suprarenal in diseases of the eye since 1894. Continued use of it from that time, with increasing gratification, enables him to define its advantages and limitations in diseases of the eye, ear, nose, and throat. The method of its preparation Bates published in the New York *Medical Journal*, March, 1896, but briefly it consists in mixing 10 grains of the dried gland with two drachms of water and filter. "The filtrate is a one per cent. solution of the extract." The active principle of the gland is incompatible with preservatives, and is weakened by heat. The aqueous extract of the suprarenal gland is the most powerful astringent known. The pupil is not affected when it is used in the eye, and it has no antiseptic or anæsthetic properties. The extract will whiten the granulations which are found in cases of suppurative otitis. In dry catarrh the extract is often valuable to relieve congestion. Cases of tinnitus have been permanently relieved by the suprarenal extract, and it will lessen the congestion of the turbinated bodies after cocaine and other astringents fail. Cocaine and the extract are incompatible, but may be used alternately.

Another communication on this preparation comes from H. L. Swain,[16] of New Haven, of which the following were the conclusions: (1) The aqueous extract of suprarenal glands was a powerful, local, vasoconstrictor agent, and a contractor of erectile tissue, which it was safe to use in very considerable amounts without dangerous or deleterious effects locally or to the general constitution of the individual; (2) these local effects could be reproduced in the same individual, apparently any number of times, without entailing any vicious habit to either the tissue or the individual; (3) the use of the extract seemed rather to heighten the effects which might be expected from any given drug used locally after it; (4) in acute congestions it had its widest application and greatest opportunity for good, but in certain chronic conditions of the hay-fever type, when redundant tissue seemed prone to develop, it could be relied upon as one of the most helpful adjuvants at command.

[14] Amer. Jour. Med. Sci., Nov. 9, 1898; Res. Hebd. de Laryng. [15] New York Med, Rec., Oct. 8, 1893. [16] Amer. Med. Surg. Bull., July 10; 1898; Med. Rec., Vol. LVII., p. 819.

### GENITO-URINARY SURGERY.

**Vicarious Urination.**—A remarkable case of vicarious urination is recorded, in which the secretion of the bladder gradually diminished accompanied by a copious secretion of urinous fluid from the lower limbs between the knee and ankle.

**Kidney.**—In cases of uncertainty of diagnosis on exploration it is sometimes valuable to resect a small piece of the diseased kidney for microscopical and bacteriological examination. Bloch refers to seven cases, in which such practice has been useful in his opinion, and he holds that such a procedure promotes the conservative surgery of the organ.[1] The statistics of nephrectomy for pyelonephritis are very bad, but Coelho has had the good fortune to have three successful results, although he acknowledges the risks and severity of the operation.[2] The statistics, pathology and treatment of nephrolithiasis are reviewed by Dr. Charles S. Cumston (Boston). His paper is chiefly useful for the historical and bibliographical references.[3]

**Congenital Malformations** of the ureters have been discussed in the *Practitioner*.[4] Reference is made to eighteen cases, published by Sutherland and Edington, including seven cases of congenital stenosis of the ureter. Two cases are figured, representing stenosis of the ureter, near the kidney and also close to the bladder.

**Traumatic Lesions** of the ureters have received much attention of late, both at home and abroad. The methods of suture have been described by Mr. Henry Morris in his Hunterian Lectures,[5] who mentions the possibility of stretching the ureter. Various sutures have been used with success, so that the probabilities are that any reasonable method may be employed. It is usual to provide a peritoneal covering for the part sutured, which is thereby rendered more secure. When the ureter is divided close to the bowel, in which place it would be difficult to suture, the lower end may be closed and the upper end be implanted into the bladder and stitched thereto.

**Ureteral Calculi** may be excised when accessible, the ureter being subsequently sutured. It may be necessary to expose it by an incision as for the common iliac vessels. When the stone is low down, close to the bladder, it has been recommended to explore through the perineum in the male or vagina in the female, and a successful case has been recorded of operation on these lines.[6]

**Bladder.**—Interesting observations on the bladder epithelium have been made by Dr. Percy Dawson.[7] The patient had slight urethritis and epididymitis for which intravesical injections of potassium permanganate were ordered. He retained these in the bladder, after which epithelium came away in large flakes, which afforded opportunities for careful histological examination. The surface cells are of very variable size and irregular polygonal shape, the outline resembling that of "prickle" cells. The protoplasm is usually marked by numerous fine vacuoles, occasionally the greater part of the interior is a vacuole. The most striking feature is the budding of the nuclei. These buds appear to arise by gemmation from the mother nucleus. Other cells are seen which are multinucleated and the inference is that they arise by a similar process.

[1] Revue de Chirurgie, June, 1898. [2] Amer. Jour. Med. Sci., Aug., 1898. [3] Internat. Med. Mag., July, 1898. [4] Carless, Practitioner, July, 1898. [5] Brit. Med. Jour., April 2, 1898. [6] Edinburgh Med. Jour., Mar., 1898. [7] Johns Hopkins Bulletin, July, 1898.

